# Detection of *Mycobacterium tuberculosis* in Sputum by Gas Chromatography-Mass Spectrometry of Methyl Mycocerosates Released by Thermochemolysis

**DOI:** 10.1371/journal.pone.0032836

**Published:** 2012-03-05

**Authors:** Denise M. O'Sullivan, Simona C. Nicoara, Reggie Mutetwa, Stanley Mungofa, Oona Y-C. Lee, David E. Minnikin, Max W. Bardwell, Elizabeth L. Corbett, Ruth McNerney, Geraint H. Morgan

**Affiliations:** 1 Department of Pathogen Molecular Biology, London School of Hygiene & Tropical Medicine, London, United Kingdom; 2 Department of Physical Sciences, The Open University, Milton Keynes, United Kingdom; 3 Biomedical Research and Training Institute, Harare, Zimbabwe; 4 School of Biosciences, University of Birmingham, Edgbaston, Birmingham, United Kingdom; French National Centre for Scientific Research - Université de Toulouse, France

## Abstract

Tuberculosis requires rapid diagnosis to prevent further transmission and allow prompt administration of treatment. Current methods for diagnosing pulmonary tuberculosis lack sensitivity are expensive or are extremely slow. The identification of lipids using gas chromatography- electron impact mass spectrometry (GC-EI/MS) could provide an alternative solution. We have studied mycocerosic acid components of the phthiocerol dimycocerosate (PDIM) family of lipids using thermochemolysis GC-EI/MS. To facilitate use of the technology in a routine diagnostic laboratory a simple extraction procedure was employed where PDIMs were extracted from sputum using petroleum ether, a solvent of low polarity. We also investigated a method using methanolic tetramethylammonium hydroxide, which facilitates direct transesterification of acidic components to methyl esters in the inlet of the GC-MS system. This eliminates conventional chemical manipulations allowing rapid and convenient analysis of samples. When applied to an initial set of 40 sputum samples, interpretable results were obtained for 35 samples with a sensitivity relative to culture of 94% (95%CI: 69.2,100) and a specificity of 100% (95%CI: 78.1,100). However, blinded testing of a larger set of 395 sputum samples found the assay to have a sensitivity of 61.3% (95%CI: 54.9,67.3) and a specificity of 70.6% (95%CI: 62.3,77.8) when compared to culture. Using the results obtained we developed an improved set of classification criteria, which when applied in a blinded re-analysis increased the sensitivity and specificity of the assay to 64.9% (95%CI: 58.6,70.8) and 76.2% (95%CI: 68.2,82.8) respectively. Highly variable levels of background signal were observed from individual sputum samples that inhibited interpretation of the data. The diagnostic potential of using thermochemolytic GC-EI/MS of PDIM biomarkers for diagnosis of tuberculosis in sputum has been established; however, further refinements in sample processing are required to enhance the sensitivity and robustness of the test.

## Introduction

Tuberculosis (TB) is a global health problem. The World Health Organization estimated 9.4 million incident cases during 2009 and 1.7 million deaths [1]. The challenge in reducing the global burden of tuberculosis is rapid diagnosis followed by appropriate treatment. Current methods of diagnosing TB are inadequate. The most widely used test, microscopic examination of sputum, lacks sensitivity and global cases detection rates remain low [2]. It is estimated that only 63% of incident cases are detected each year [1]. Definitive diagnosis requires isolation and culture of the bacilli which may take weeks and requires considerable laboratory infrastructure to protect against infection [2], [3].

**Figure 1:**
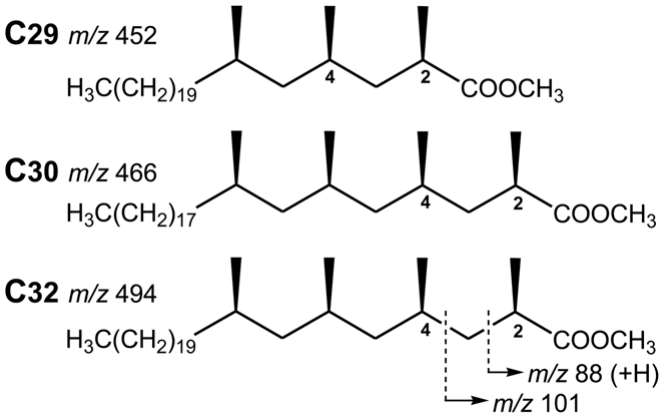
Structures of the *M. tuberculosis* C_29_, C_30_ and C_32_ mycocerosic acid methyl esters. The ions at *m/z* 452, 466 and 494 correspond to the molecular weights of these esters. Ions at *m/z* 88 and 101 are characteristic for 2-methyl branched fatty acid methyl esters, the former resulting from a well-known “McLafferty” rearrangement with transfer of one hydrogen atom.

Detection of lipid moieties, which are essential components of the cell envelope of *Mycobacterium tuberculosis*, offers an alternative approach for diagnosis. Chemical analysis has the advantage over culture methods in that it can be rapid and does not require live organisms. The mycocerosic acid components of the phthiocerol dimycocerosate (PDIM) family of complex lipids are established diagnostic markers for tuberculosis [4], [5]. Fatty acids of this type are found in *M. tuberculosis, Mycobacterium bovis, Mycobacterium kansasii, Mycobacterium leprae, Mycobacterium marinum, Mycobacterium gastri, Mycobacterium haemophilum* and *Mycobacterium ulcerans* [6], [7], [8]. Members of the *M. tuberculosis* complex have a characteristic profile composed of trimethyl C_29_ and tetramethyl C_30_ and C_32_ mycocerosates (Figure 1), while the other related mycobacteria mentioned have distinct profiles [4], [9]. Analysis of mycocerosates has previously been performed using gas chromatography (GC) followed by electron capture detection or negative ion chemical ionisation MS [4], [5], [9], [10], including detection in sputum [5], [10].

*M. tuberculosis* PDIMs are highly stable waxes, composed of mixtures of long-chain multimethyl-branched mycocerosic acids esterified to C_34_ and C_36_ long-chain diols, the phthiocerols ([4], [8]). Degradation of these extremely hydrophobic waxes requires strong chemical methods. Alkaline hydrolysis requires an overnight non-aqueous potassium hydroxide/methanol/toluene treatment system [4], [9]. Alternatively, the PDIMs may be cleaved by reduction [7], [11], but such a procedure is complex and lengthy and unattractive for rapid detection or use in a clinical diagnostic laboratory.

An advantage of the PDIM lipids over other lipid entities is their exceptionally low polarity, which enables them to be preferentially extracted by non-polar solvents [10], [12], separating them from the majority of the other mycobacterial and mammalian lipids. We have investigated a simple extraction procedure followed by thermally assisted hydrolysis and methylation (THM), prior to the analysis by gas chromatography (GC) coupled with electron impact mass spectrometry (EI/MS), to ascertain the potential for a rapid diagnostic test for pulmonary tuberculosis based on detection of the mycocerosic acid components of PDIMs. This protocol involves heating samples with methanolic tetramethylammonium hydroxide (TMAH), which facilitates direct transesterification of the acidic components to methyl esters [13]. Performing this transformation in the programmable temperature vaporization (PTV) inlet of a GC-MS eliminates conventional chemical manipulations allowing rapid analysis of clinical samples. The novelty of this work is the application of THM-GC-EI/MS to the analysis of PDIMs extracted from sputum.

## Results

**Figure 2:**
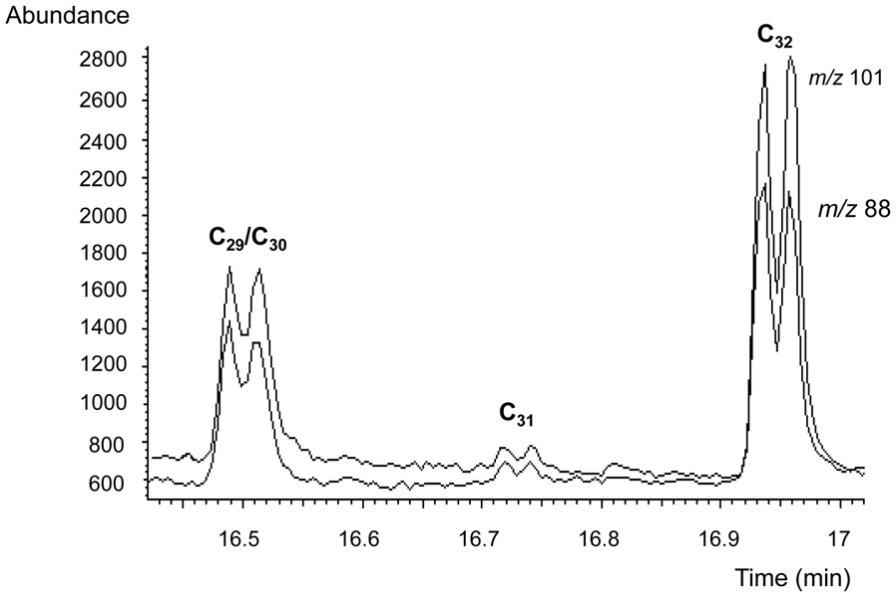
Thermochemolytic-gas chromatography-electron impact/mass spectrometry (THM-GC-EI/MS) of purified phthiocerol dimycocerosate (PDIM) waxes. The selected ion monitored EI/MS chromatogram shows the target fragment ions *m/z* 101 and *m/z* 88, resulting from the major trimethyl C_29_ and tetramethyl C_30_, and C_32_ mycocerosates released by thermochemolysis of the PDIMs. A minor proportion of a tetramethyl C_31_ mycocerosate is also indicated. The characteristic doublet peaks are due to racemisation during the alkaline hydrolysis. The method relied on recognizing comparable proportions of the combined C_29_/C_30_ peak and the C_32_ peak, as was the case in all the positive specimens. Some minor variation in the ratios of the individual peaks was observed, but this did not affect the diagnostic ratios.

Thermochemolytic-gas chromatography-electron impact mass spectrometry (THM-GC-EI/MS) of standard phthiocerol dimycocerosate (PDIM) waxes generated profiles characteristic of mycocerosic acid methyl esters from *M. tuberculosis*. The diagnostic mycocerosates are trimethyl C_29_ and tetramethyl C_30_ and C_32_ acids [4], [9]. As shown in Figure 2, each mycocerosate is represented as a characteristic double peak due to racemisation during the alkaline hydrolysis [4]. As explained in the discussion section, the earlier eluting double peak represents overlapping trimethyl C_29_ and tetramethyl C_30_ mycocerosates; the additional methyl branch in the larger C_30_ mycocerosate increases its volatility so that it co-elutes with the smaller C_29_ component. The later eluting double peak corresponds to the two diastereoisomers of tetramethyl C_32_ mycocerosate.

### Spiking of negative sputum with PDIMs standard

**Figure 3:**
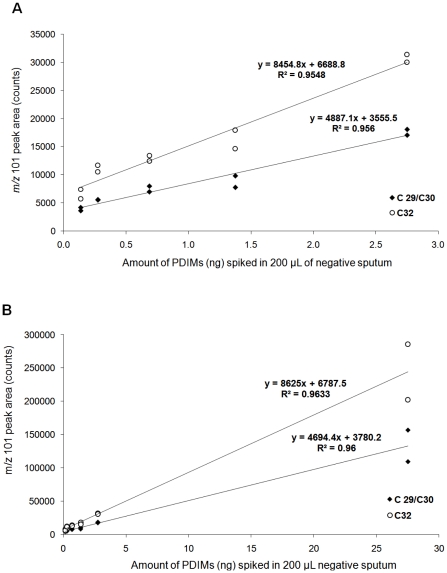
THM-GC-EI/MS of sputum spiked with PDIMs. The graphs show the peak areas obtained for C_29_/C_30_ and C_32_ mycocerosates generated from spiking 0.2 mL of pooled TB negative sputum samples with PDIM standard, (A) in the range 0.14–2.75 ng, and (B) expanding the range between 0.14–27.5 ng.

Sputum samples were processed using sodium hydroxide in a standard decontamination procedure routinely applied prior to *M. tuberculosis* culture [14]. Apolar lipids were extracted from samples with petroleum ether 60–80°C (PE) using a modified method from Dobson *et al.*. Duplicate analyses of extracts obtained from negative sputum samples spiked with PDIMs standard (140–27,500 pg/mL) demonstrated that the yield of the mycocerosic acid methyl esters was proportional to the amount of material added, within the range tested (Figure 3). Both the C_29_/C_30_ and C_32_ peaks were clearly visible in the extract spiked at the lowest level analyzed, 140 pg of PDIMs (equivalent to 7 pg of PDIMs loaded in liner), with an average signal to noise ratio (S/N) of 9.1 for C_29_/C_30_, and of 39.3 for C_32_.

### Spiking of negative sputum with culture

**Figure 4:**
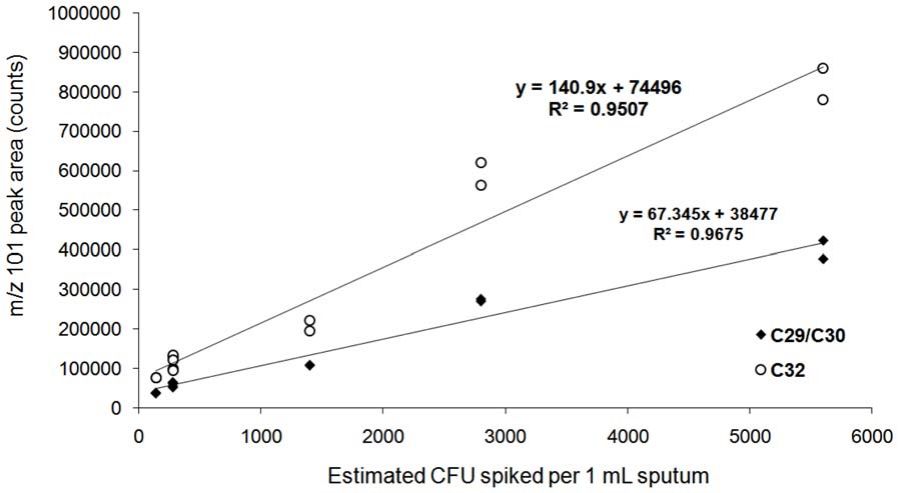
THM-GC-EI/MS of sputum spiked with *M. tuberculosis* bacilli. The graph shows the peak areas obtained for C_29_/C_30_ and C_32_ mycocerosates generated from spiking 1 mL of pooled TB negative sputum sample with *M. tuberculosis* culture (140–5,600 CFU/mL).

**Figure 5:**
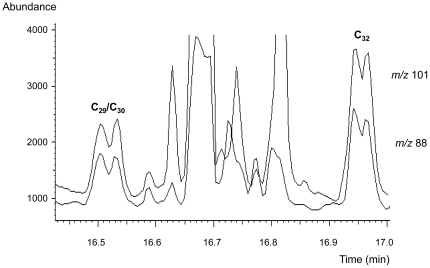
THM-GC-EI/MS traces of TB negative sputum spiked with *M. tuberculosis* bacilli. The chromatograms show the fragment ions *m/z* 101 and *m/z* 88 for the extract from 1 ml sample of TB negative sputum spiked with 140 CFU of *M. tuberculosis* culture.

Duplicate analyses of the extracts obtained from pooled negative sputum samples spiked with *M. tuberculosis* culture (140–5,600 CFU per mL) indicated that the areas of the mycocerosic acid methyl esters peaks showed a linear relationship with the number of bacteria in the range tested (Figure 4). As can be seen in Figure 5, both the C_29_/C_30_ and C_32_ peaks were clearly visible in the extract spiked at the lowest level analyzed 140 CFU per mL, and had an average signal to noise ratio (S/N) of 36.2 for C_29_/C_30_, and 55.3 for C_32_.

### Identification of mycocerosic acids in clinical isolates

**Figure 6:**
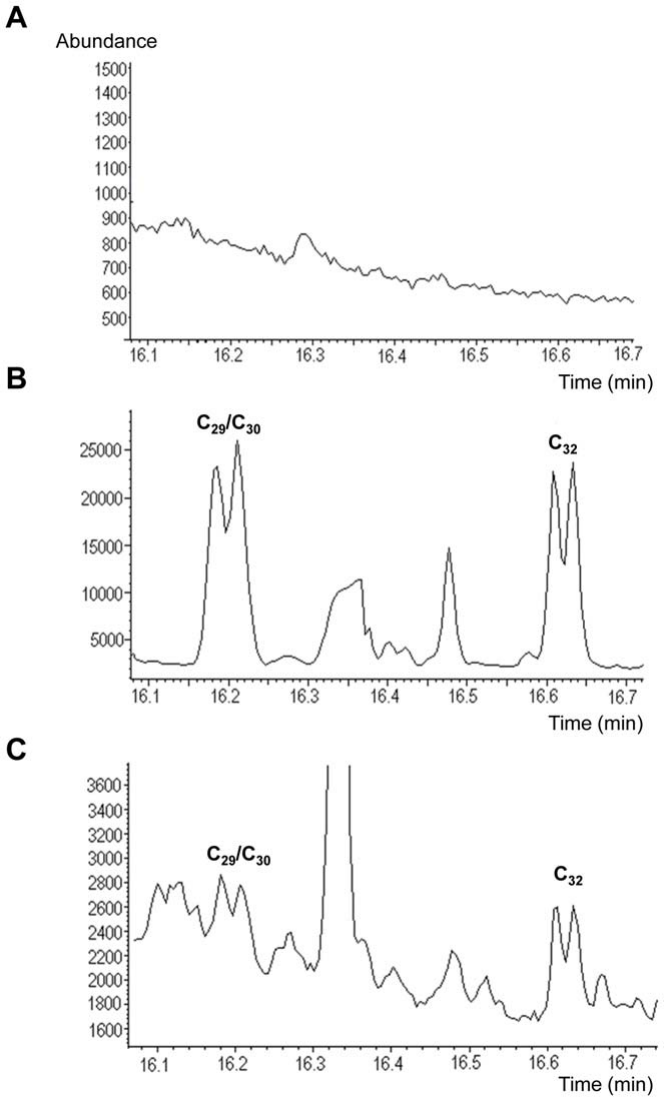
Examples of THM-GC-EI/MS chromatograms from sputum specimens from suspected TB cases. Chromatograms show the profile of the fragment ion *m/z* 101 in (A) negative, and positive sputum samples with (B) high, and (C) low amounts of PDIMs present. Note the characteristic doublets for the mycocerosic acid methyl esters C_29_/C_30_ and C_32_ in b–c.

Sputum samples from suspected TB cases were processed in two batches; the first panel (the test batch) consisted of 40 samples (20 smear positive culture positive and 20 smear and culture negative), and the second panel (the evaluation batch) consisted of 395 samples. All samples were subjected to smear microscopy and culture for *M. tuberculosis* prior to the THM-GC-EI/MS analysis. On blinded testing the panel of 40 sputum samples, mycocerosic acids were identified in 14/20 smear and culture positive sputum specimens. Three smear and culture positive sputum specimens were incorrectly assigned as negative and the remaining three were assigned as indeterminate. Of the smear and culture negative sputum specimens, 18/20 were assigned as negative and the remaining 2/20 were classified as indeterminate. Following un-blinding, the characteristic doublet peaks were identified at the appropriate retention times, in two of the false negatives. They were initially miss-classified owing to their low abundance within a high matrix background. When the 5 undetermined samples were excluded from the analysis, the unblinded results obtained demonstrated a sensitivity of 94% (95%CI: 69.2,100) and a specificity of 100% (95%CI: 78.1,100). Figure 6 shows example chromatograms for (a) a negative sputum sample, and two positive sputum samples with a (b) high and (c) low level of PDIMs. A further 395 sputum samples were then analyzed in a blinded fashion by THM-GC-EI/MS, based on initial classification criteria, namely the presence of doublets in the *m/z* 101 and *m/z* 88 chromatograms at the expected retention times. Results are presented in Table 1. Four samples were not determined by THM-GC-EI/MS and were excluded from further analysis as their chromatograms were uninterpretable due to overloading of the column. Of the remaining 391 samples THM-GC-EI/MS detected 68.8% (95%CI: 61.9,75) of smear positive culture confirmed cases and 25.6% (95%CI: 13.9,41.5) of smear negative cases. Sensitivity for detection of culture confirmed TB cases was 61.3% (95%CI: 54.9,67.3) and the specificity was 70.6% (95%CI: 62.3,77.8).

**Table 1:** Performance of THM-GC-EI/MS compared to smear microscopy and culture on Lowenstein-Jensen for detection *M. tuberculosis* in 395 sputum samples.

	Culture positiveSmear positive	Culture positiveSmear negative	Culture negative	Total samples
**THM-GC-EI/MS positive**	141	11	42	194
**THM- GC-EI/MS negative**	64	32	101	197
**Indeterminate** #	1	1	2	4
**Total samples**	206	44	145	395

# Four samples were not determined due to overloading of the GC column and were excluded from further analysis.

Following unblinding, chromatograms were re-studied to evaluate where errors had been made and whether lessons could be learnt about their misclassification. False negatives were identified where small characteristic doublet peaks had been missed within a high background. False positives were identified where instead of a doublet, two closely eluting peaks had actually been observed at, or close to, the anticipated retention times, or where doublets appeared but not at the in correct retention times. To provide a more careful assessment of chromatograms, revised classification criteria where drawn up, focusing on a more stringent approach to the chromatographic alignment, and to strictly matching retention times and the doublets profiles with the daily standard runs.

A positive result was assigned to a chromatogram showing the following features:

- the presence of the characteristic doublets in both *m/z* 101 and *m/z* 88 traces, at the retention times for both C_29_/C_30_ and C_32_, respectively;
- the time interval between the two characteristic doublets in the sputum sample to rigorously match that in the PDIM standard daily run;
- the doublet peak at C_29_/C_30_ to be comparable in size to the doublet peak at C_32_, in both ion chromatograms *m/z* 101 and *m/z* 88;
- both the *m/z* 101 and *m/z* 88 traces closely matching each other in shape and size.

A negative result was assigned to the following features:

- the absence of one or both of the characteristic doublets at the expected retention time in the *m/z* 101 and *m/z* 88 ion chromatograms;
- the presence of a single peak at either of the expected retention times in the *m/z* 101 and *m/z* 88 ion chromatograms
- the presence of two closely eluting, but distinct peaks, instead of doublets, at either of the expected retention times in the *m/z* 101 and *m/z* 88 ion chromatograms;
- a time interval between the suspected C_29_/C_30_ and C_32_ signals not matching rigorously that in the daily standard run;
- the doublets at C_29_/C_30_ and at C_32_ are not comparable in size
- the traces of *m/z* 101 and *m/z* 88 ion chromatograms do not match each other in shape and size.

The chromatograms were then re-blinded and examined by a newly trained analyst, with no prior contact with the data. Data obtained are presented in Table 2. This approach resulted in the detection of 74.1% (95%CI: 67.5,79.9) of smear positive culture confirmed cases and 20.9% of smear negative cases (95%CI: 10.6,36.5). Its overall sensitivity relative to culture was 64.9% (95%CI: 58.6,70.8) and the specificity was 76.2% (95%CI: 68.2,82.8).

**Table 2:** Secondary analysis of the performance of THM-GC-EI/MS compared to smear microscopy and culture on Lowenstein Jensen.

	Culture positive Smear positive	Culture positiveSmear negative	Culture negative	Total samples
**THM-GC-EI/MS positive**	152	9	34	195
**THM-GC-EI/MS negative**	53	34	109	196
**Indeterminate** #	1	1	2	4
**Total samples**	206	44	145	395

A refined algorithm for interpreting the THM-GC-EI/MS chromatograms, developed by using knowledge from an initial analysis of 395 samples, was applied in a second round of blinded analysis by a newly trained operator.

# Four samples were not determined due to overloading of the GC column and were excluded from further analysis.

## Discussion

This study has demonstrated the use of mycocerosic acids as a biomarker for diagnosis of tuberculosis disease from sputum. In principle, the method involves preferential extraction of the apolar PDIM waxes into PE solution and the PDIMs are then heated with methanolic TMAH in the inlet of the GC-MS instrument. This alkaline reagent hydrolyzes the PDIMs to yield tetramethylammonium salts of the mycocerosic acids, which pyrolyse to produce methyl mycocerosates with the molecular formulae shown in Figure 1 [13]. For fatty acids with methyl branches in the 2- and 4-positions the alkaline hydrolysis randomizes the natural configuration at position 2 in a process known as “racemisation”. The configuration at position 4 is not disturbed, so the interaction between the methyl branches at positions 2 and 4 can occur in two ways, producing physically distinct compounds, termed “diastereoisomers”. These components are readily separated on the GC column (Figure 2); for example, the first C_32_ mycocerosate peak is the natural acid followed by similar proportions of the racemised acid. Such double peaks are a diagnostic feature for 2,4-dimethyl-branched fatty acids, such as the mycocerosates (Figure 1). The two peaks for the C_29_/C_30_ mycocerosate are actually two overlapping doublets for each separate mycocerosate; the additional methyl branch in the larger C_30_ mycocerosate increases its volatility so that it co-elutes with the smaller C_29_ component.

In the bacteria culture studied, methyl mycocerosates were detected in PE extracts from sputum samples spiked at a level of 140 CFU mL^−1^, suggesting a sensitivity considerably greater than that of smear microscopy which is estimated to require 10^4^ CFU mL^−1^ [15], [16]. When testing the sputum samples it was necessary to concentrate the sputum deposit and extract the samples. We applied the standard decontamination procedures used in diagnostic culture laboratories and heat treated samples to sterilize and allow safe handling in the open laboratory. We then applied a simple extraction of the apolar lipids prior to analysis by THM-GC-EI/MS. Initial analysis of 40 sputum samples taken from TB suspects with well defined clinical and bacteriological outcomes provided interpretable results from 35 (87.5%) samples. Following unblinding of the microbiological data, improved assignment was possible and results were obtained from 92.5% of samples tested with a further 2 samples identified as THM-GC-EI/MS positives, suggesting that high sensitivity was achievable. When the assay was evaluated with a larger set of sputum specimens in a blinded fashion, interpretable THM-GC-EI/MS results were obtained for 391/395 (99%) of samples. However, the initial performance of the assay demonstrated reduced sensitivity of 61.3% and reduced specificity of 70.6%. Examination of unblinded results from the 395 samples made it possible to enhance the classification criteria for interpreting the THM-GC-EI/MS chromatograms. When mycocerosates are present well above the matrix then a strong pair of doublets signal is generated and the above decision process is straight forward, similarly for negative samples which have a clean chromatogram. However, for samples where results are not visually obvious a more rigorous method of assignment was developed. Training of a new operator using the refined algorithm resulted in an improved sensitivity and specificity of 64.9 and 76.3% respectively. Although a proportion (20.9%) of smear negative culture positive cases were identified the detection rate for smear positive cases of less than 75% is considered suboptimal for the diagnosis of tuberculosis. It should be noted that the smear positivity rate of 82.7% of culture confirmed cases observed in this study is usually high for a sub Saharan African setting where co-infection with HIV is common. In Zimbabwe during 2009, the estimated smear positivity rate in new pulmonary cases was 29% [1]. Following unblinding of the microbiological results, further analysis suggested that the performance of the THM-GC-EI/MS assay was being compromised due to the high background signal in some samples. This was highly variable between samples and may have been due to compounds of bacterial or human origin. High levels of matrix compounds affect the performance of the GC-MS instrument and its ability to separate compounds at trace levels, also causing column overloading and blockages. In addition to overloading the analytical capacity for individual samples, the buildup of contamination within the injector, column and MS may occur, necessitating periodic cleaning if large numbers of samples are to be processed. To improve sensitivity, further optimization of the sample preparation would be required to produce a cleaner sample. Despite this, we have shown that THM-GC-EI/MS can detect mycocerosic acids present in clinical sputum samples. The time of the analysis was approximately two hours per sample and sample handling was minimized by the use of online hydrolysis and methylation. An attempt to use thermochemolysis for the detection of *M. tuberculosis* directly in sputum, has been described previously where the target was tuberculostearic acid (TBSA) [17]. Mycocerosic acids are a more suitable diagnostic biomarker for *M. tuberculosis* than TBSA. Firstly, TBSA is less specific and present in the bacteria belonging to the Actinomycetales which include *Nocardia* and most mycobacteria. Secondly, TBSA is found in several components in the cell membrane, which requires complicated chemical extraction methods to release it, whereas PDIM waxes have the advantage of being selectively extracted from decontaminated sputum samples. Thirdly, TBSA is difficult to identify because its branched C_19_ structure makes it hard to separate it from stearic acid (C_18_) and similar acids; techniques, such as column switching multi-dimensional GC, may be necessary [5]. The mycocerosic acid methyl esters separate in an area of the chromatogram where there are fewer other compounds present and are more easily observed because the C_29_, C_30_ and C_32_ components appear as two characteristic doublet peaks. An alternative GC-MS method for detecting mycocerosic acids uses negative-ion chemical ionization (NICI) of pentafluorobenzyl esters in sputum [10]. This method requires separate hydrolysis and derivatisation which is slow and laborious. Similarly, another complicated process, involving PDIMs isolation and hydrolysis, permitted detection of sputum mycocerosates by electron capture GC [5].

In order for an assay to be deployable in a routine diagnostic laboratory, the extraction required must be simple, and the analysis rapid and robust. Direct analysis of the sputum may be performed using THM-GC-EI/MS (data not shown) but the heterogeneity of the sample and high levels of background contamination discourages this approach. The application of liquid/liquid extraction of apolar lipids is attractive due to its simplicity and potential for automation but our data suggest that further clean up is necessary. Solid phase extraction (SPE) of the PDIMs may improve sample quality and reduce the burden on the analytical instrument from the matrix compounds. When compared to culture THM-GC-MS methods offer rapid testing with the potential to provide ‘same day’ analysis. They also reduce the necessity to handle infectious materials. Amplification of nucleic acids offers an alternative rapid test system. The recently launched Xpert MTB/RIF assay (Cepheid, Sunnyvale, USA) allows sensitive detection of *M. tuberculosis* DNA in less than two hours [18], [19]. However, the anticipated running costs are substantial: approximately USD 21 per test in the public sector of TB endemic countries [20] and considerably higher in the private sector or industrialized countries. Should it prove sufficiently sensitive and robust, through sample extraction optimization, then THM-GC-EI/MS may provide a cost effective alternative. However, the high cost of the instrumentation and use of organic solvents may limit application in developing country laboratories.

## Materials and Methods

### Ethical approval

The study and collection of samples at the Beatrice Road Infectious Diseases Hospital was reviewed and approved by the National Ethics Committee of the Medical Research Council of Zimbabwe, Harare, Zimbabwe, the Institutional Review Board of Biomedical Research and Training Center, Harare, Zimbabwe, and the Research Ethics Committee of the London School of Hygiene and Tropical Medicine, London, UK. Informed written consent was obtained from all patients who enrolled in the study.

### PDIM standards

The PDIM waxes were extracted and purified from *M. tuberculosis* strain C [11] according to the procedures described by Dobson *et al.* [12]. In essence, non-polar lipids were extracted from freeze-dried biomass using a biphasic mixture of petroleum ether and aqueous methanol. The main component of the PDIMs, based on phthiocerol A [8], was purified from the petroleum ether extract by preparative thin-layer chromatography [7].

### *M. tuberculosis* culture

*M. tuberculosis* H37Rv (laboratory strain) was cultured in 10 mL Middlebrook 7H9 broth, containing 0.2% Tween 80 (BDH Becton Dickinson Diagnostic Systems, Sparks, MD) and 10% albumin dextrose catalase enrichment (Becton Dickinson Diagnostic Systems, Sparks, MD) at 37°C for 10 days. Following this, cells were harvested and re-suspended in sterile distilled water. A Miles and Misra plate count was performed [21] and the colony forming units (CFU) were estimated.

### Clinical Samples

Sputum samples were collected from TB patients and suspects presenting at Beatrice Road Infectious Diseases Hospital in Harare, Zimbabwe as described by Mutetwa *et al.* [22]. Sputum specimens were homogenized by vortexing with 4 mm beads, and then split into two; one sample was used for smear microscopy and culture. Fluorescent smear microscopy (auramine O) was undertaken with positive samples confirmed by Ziehl-Neelsen (ZN) staining. Positive cultures on Lowenstein Jensen were confirmed as *M. tuberculosis* complex by MPB64 antigen detection (Capillia™, Becton Dickinson, Sparks, MD, USA). The second aliquot of sputum was stored frozen prior to shipment to London where they were stored at −80°C prior to analysis by THM-GC-EI/MS. The allocation of aliquots to either process was randomized to control operator bias. Clinical and microbiological data were not revealed to those undertaking the PDIMs analysis.

Sputum samples were processed in two batches; the first panel (the test batch) consisted of 40 samples (20 smear positive culture positive and 20 smear and culture negative), and the second panel (the evaluation batch) consisted of 395 samples. Samples were processed using a standard protocol routinely applied to sputum prior to culture [14]. In brief, 0.5 mL of 4% sodium hydroxide was added to 0.5 mL of each sample. Following mixing, samples were left to stand for 20 min at room temperature with periodic vortexing, after which an equal volume of neutralization buffer was added (140 g/L potassium phosphate monobasic and 8 mL 1% phenol red). The mixtures were centrifuged at 3000 *g* for 30 min. The supernatants were decanted and the pellets re-suspended in 10 mL sterile distilled water. Centrifugation was repeated and the supernatants decanted leaving behind deposits (approximately 1 mL). The deposits were heated in a water bath at 100°C for 30 min, in order to sterilize the samples. Deposits were further concentrated by centrifugation at 14,000 *g* for 15 min, leaving 0.2 mL deposits for subsequent apolar lipid extraction.

### Apolar Lipid Extraction

The apolar lipids from dilutions of *M. tuberculosis* culture and sputum specimens were extracted using a modified method from Dobson *et al.* [12]. To 0.2 mL of deposit, 1.8 mL methanol and 1 mL petroleum ether 60–80°C (PE) were added and mixed on a tube rotator for 15 min. After centrifugation at 1,200 *g* for 1 min, the upper PE layer, containing the apolar lipids, was removed and stored at 4°C.

Prior to analyzing the sputum samples, the performance of the thermochemolysis and the extraction of PDIMs were evaluated using samples spiked with PDIMs or bacteria harvested from culture. Aliquots of negative pooled sputum (0.2 mL) were spiked with a PDIMs standard (140–27,500 pg of total PDIMs). Each spiked sample was vortexed for 30 seconds and then the apolar lipids were extracted, following the procedure described above. Assuming an extraction efficiency of 100%, then this would be equivalent to a concentration range of PDIMs between 140–27,500 pg/mL of petroleum ether. Hence the total quantity of PDIMs analysed ranged between 7–1,375 pg/liner. *M. tuberculosis* culture (140–5,600 CFU/mL) was spiked into 1 mL aliquots of negative pooled sputum. Each sample was vortexed and centrifuged at 14,000 g for 15 min. The supernatant was discarded, leaving behind 0.2 mL deposit for apolar lipid extraction.

### Thermochemolysis and GC-EI/MS analysis

The equipment used was an Agilent 7890A GC coupled to a 5975C quadrupole MS, with an electron impact (EI) ionization source set at 70 eV impact energy and 35 µA emission current. A PAL-CTC autosampler, modified with a LINEX liner exchanger, was used in conjunction with an Optic3 programmable temperature vaporization (PTV) inlet. Testing of sputum extracts was performed blinded with THM-GC-EI/MS operators not aware of the microbiological and clinical data.

Fifty microlitres of the PE extract or of the PDIM solutions were manually applied to a quartz wool plug inside the Optic glass liner and dried at 60–70°C for 10 min. The liners were then loaded into the autosampler. Following automated injection of 50 µL methanolic tetramethylammonium hydroxide (TMAH) (12.5%), derivatization of lipids was performed in the Optic3 PTV inlet at 380°C. The GC analysis was carried out on a DB-5MS capillary column (15 m×0.25 mm×0.25 µm) (Agilent Technologies, UK) with a temperature program of 50°C (8 min) to 350°C (1 min) @ 30°C/min, and a carrier gas flow rate of 1.4 mL of helium per min.

The mass spectrometer was operated in selected ion monitoring (SIM) mode. The target compounds were detected by monitoring for the largest fragment ions at *m/z* 88 and 101, which are characteristic for all derivatised mycocerosates [10], and the molecular ions *m/z* 452 (C_29_), 466 (C_30_) and 494 (C_32_) (Figure 1). Quantification of the mycocerosic acid methyl esters was performed by measuring the areas of the target peaks in the chromatogram for the *m/z* 101 fragment ion. The quantitative analyses are with respect to the total amount of PDIMs present which includes the abundance of the C_29_/C_30_ and C_32_ peaks. Throughout this paper, all the data analysis and interpretation were done based on visual examination, and by using the integration facility in Agilent ChemStation. As part of the data interpretation, the sample chromatograms were aligned with the daily run of PDIMs standard, the retention times (RT) were examined for chromatographic drifting, and were corrected if necessary.
